# Primary Immune Responses and Affinity Maturation Are Controlled by IgD

**DOI:** 10.3389/fimmu.2021.709240

**Published:** 2021-08-09

**Authors:** Timm Amendt, Omar El Ayoubi, Alexandra T. Linder, Gabriele Allies, Marc Young, Corinna S. Setz, Hassan Jumaa

**Affiliations:** Institute of Immunology, Ulm University Medical Center, Ulm, Germany

**Keywords:** B cell selection, IgD, antigen-valency, autoimmunity, IgM, tolerance

## Abstract

Mature B cells co-express IgM and IgD B cell antigen receptors (BCR) on their surface. While IgM BCR expression is already essential at early stages of development, the role of the IgD-class BCR remains unclear as most B cell functions appeared unchanged in IgD-deficient mice. Here, we show that IgD-deficient mice have an accelerated rate of B cell responsiveness as they activate antibody production within 24h after immunization, whereas wildtype (WT) animals required 3 days to activate primary antibody responses. Strikingly, soluble monovalent antigen suppresses IgG antibody production induced by multivalent antigen in WT mice. In contrast, IgD-deficient mice were not able to modulate IgG responses suggesting that IgD controls the activation rate of B cells and subsequent antibody production by sensing and distinguishing antigen-valences. Using an insulin-derived peptide we tested the role of IgD in autoimmunity. We show that primary autoreactive antibody responses are generated in WT and in IgD-deficient mice. However, insulin-specific autoantibodies were detected earlier and caused more severe symptoms of autoimmune diabetes in IgD-deficient mice as compared to WT mice. The rapid control of autoimmune diabetes in WT animals was associated with the generation of high-affinity IgM that protects insulin from autoimmune degradation. In IgD-deficient mice, however, the generation of high-affinity protective IgM is delayed resulting in prolonged autoimmune diabetes. Our data suggest that IgD is required for the transition from primary, highly autoreactive, to secondary antigen-specific antibody responses generated by affinity maturation.

**Graphical Abstract:**
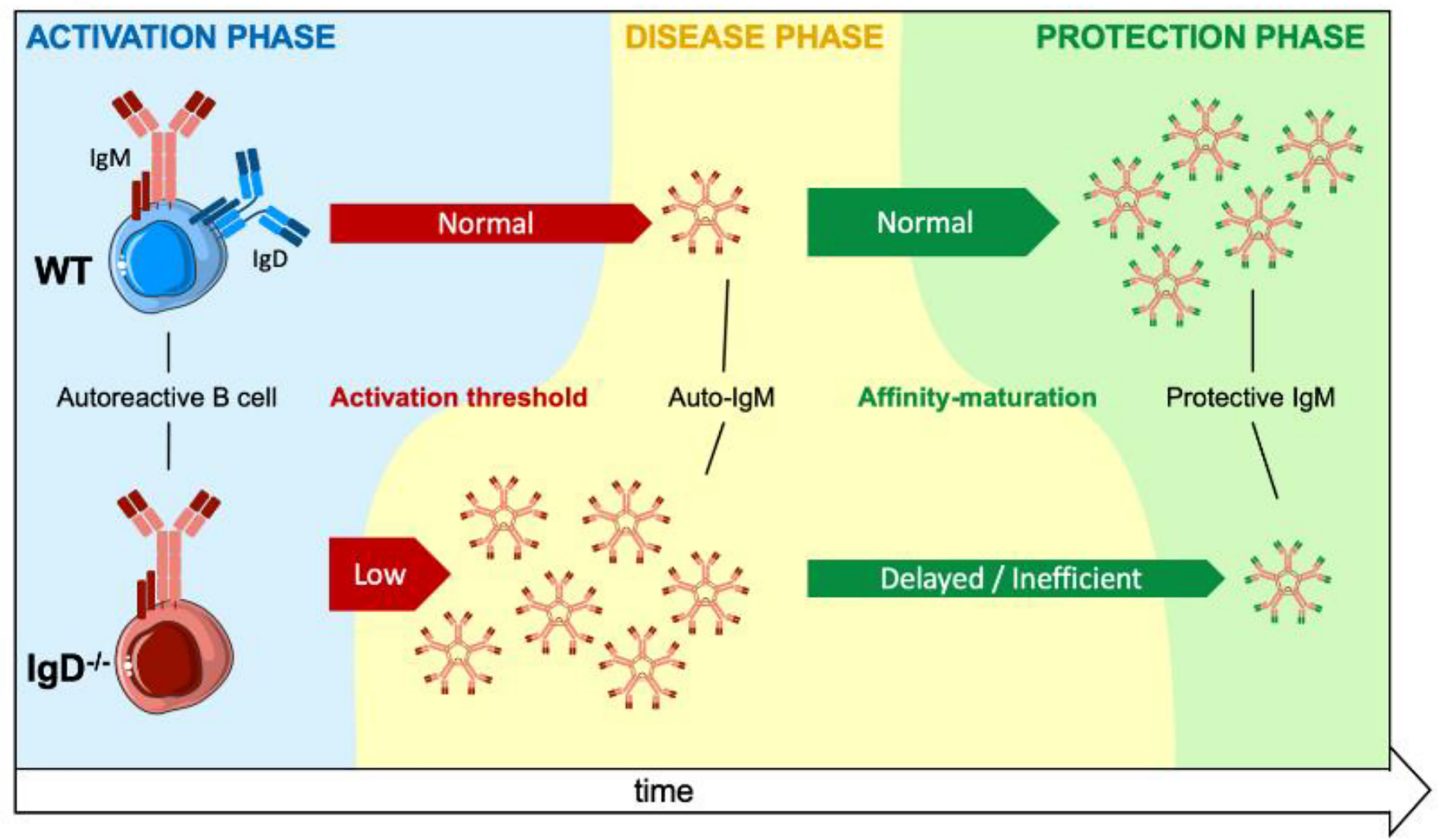


## Introduction

Maintaining physiological integrity by avoiding autoimmune reactions is achieved by self-tolerance. Currently, self-tolerance is believed to be divided into two programs. First, central tolerance that occurs in the bone marrow by deleting early self-reactive B cells or initiating receptor editing (1–4). Second, autoreactive B cells circumventing central tolerance are thought to undergo clonal anergy characterized by functional unresponsiveness and downmodulation of the IgM-class BCR (5, 6). However, these concepts can neither explain the variety of autoantibody-borne autoimmune diseases (7–9) nor provide therapeutic concepts. Importantly, the use of transgenic mouse models expressing highly affine monoclonal BCRs (1, 6) differ considerably from physiological conditions. In addition, recent studies reporting that anergic B cells from transgenic models behave like mature cells that are regulated by the antigen form and the BCR-class expressed on their membrane suggest that understanding the role of BCR-isotypes in mature B cell responsiveness is crucial for understanding B cell activation and tolerance (10, 11).

During early developmental stages, immature B cells exclusively express IgM-class BCR. Cells passing through the immature B cell stage leave the bone marrow and co-express IgM- and IgD-class BCRs sharing the same antigen-specificity but differ in the use of the heavy chain (HC) μ compared to δ. In mature B cells the IgD-class BCR becomes the predominant antigen receptor.

In the past decades, the IgD-class BCR was believed to be redundant with IgM and not involved in autoimmune processes (12–15). However, since IgD is barely detectable in human serum (16, 17) and shows structural and functional differences compared to IgM (15, 18, 19), a specialized role in B cell physiology is conceivable. Recent studies showed that IgM and IgD are associated in distinct membrane nanoclusters pairing with specific coreceptors (20, 21) and that IgD-deficient mice (12) also show delayed germinal center formation (11, 14).

Using cellular reconstitution systems or transgenic mice for hen-egg lysozyme (HEL) as model antigen, we recently reported that IgM- and IgD-class BCRs differ in their responsiveness to distinct antigen-valences. In contrast to the IgD-class BCR, IgM induces calcium flux after treatment with monovalent antigen (soluble HEL), whereas treatment with polyvalent antigen (complex HEL) activated both receptor classes. Interestingly, the presence of monovalent HEL blocked the activation of the IgD-class BCR by complex HEL due to the flexible hinge region of IgD (10, 11). Together, these data suggest a novel concept, termed adaptive tolerance and responsiveness, for the development, selection and activation of mature B-lymphocytes.

Here, we show that IgD controls the responsiveness and antibody secretion by B cells *in vivo* and that “self-tolerance” is achieved mainly by unique characteristics of immunoglobulin isotypes and by antigen-valence. Upon antigen encounter, IgD determines how fast an immune response is triggered and how long it lasts as a primary low-specific IgM response. This happens independent of whether the antigen belongs to self or non-self, which makes this IgD-mediated regulation key for the maturation of antibody responses and tolerance to self-structures.

## Methods

### Mice

8 – 15-week-old female C57BL/6 mice and IgD-deficient (12) mice were immunized intraperitoneally (i.p.) with a mixture of 50 µg antigen with 50 µg CpG-ODN1826 (Biomers) in 1x PBS. For control immunization (CI) mice received PBS and CpG-ODN1826 (50 µg/mouse). Animal experiments were performed in compliance with license 1484 for animal testing at the responsible regional board Tübingen, Germany. All mice used in this study were bred and housed within the animal facility of Ulm University under specific-pathogen-free conditions, or obtained from Charles River at 6 weeks of age. All animal experiments were done in compliance with the guidelines of the German law and were approved by the Animal Care and Committees of Ulm University and the local government.

### Peptides and Immunogens

Insulin-A-chain derived peptides (InsA) (Peptides&Elephants, Berlin) were dissolved according to their water solubility. For covalent coupling of peptides to key hole limpet hemocyanin (KLH) the N-terminal cysteine was used. The amino acid sequence used (mouse Insulin-A-chain: CSLYQLENYCN) is fully homologue to human insulin. A detailed description of the InsA autoimmunity model is provided in Amendt and Jumaa, 2021 (EMBOJ). Coupling of peptides to Streptavidin (SAV, ThermoScientific) was done by addition of biotin to the N-terminus. The C-terminus was left with an OH-group for better handling. 4-hydroxy-3-nitrophenylacetyl coupled to KLH (NP(30)KLH) or BSA (NP(15)BSA) were purchased from Biosearch Technologies.

### Flow Cytometry

Cell suspensions were Fc-receptor blocked with polyclonal rat IgG-UNLB (2,4G2; BD) and stained according to standard protocols. Detection of Biotin-conjugated peptides/antibodies were achieved by using Streptavidin Qdot605 (Molecular Probes; Invitrogen). Viable cells were distinguished from dead cells by using Fixable Viability Dye eFluor780 (eBioscience). Cells were acquired at a Canto II Flow Cytometer (BD). Plots indicate percentages in the respective gates whereas numbers in histogram plots show the mean fluorescence intensity (MFI).

### Enzyme-Linked Immunosorbent Assay (ELISA)

96-Well plates (Nunc, Maxisorp) were coated with, native Insulin (Sigma-Aldrich, Cat. 91077C), Streptavidin (ThermoScientific, Cat. 21125), calf thymus DNA (ThermoScientific, Cat.15633019), or NP-BSA (Biosearch Technologies, N-5050H-100) with 10 µg/mL, or anti-IgM, anti-IgG-antibodies (SouthernBiotech). Biotinylated peptides (2,5 µg/mL) were loaded onto SAV-coated plates in 1% BSA blocking buffer (ThermoFisher). Sera were initially diluted 1:50. Serial dilutions of 1:3 IgM or IgG antibodies (SouthernBiotech) were used as standard. The relative concentrations, stated as arbitrary unit (AU), were determined *via* detection by Alkaline Phosphatase (AP)-labeled anti-IgM/anti-IgG (SouthernBiotech), respectively. The p-nitro-phenylphosphate (pNPP; Genaxxon) in Diethanolamine buffer was added for starting of the reaction. Data were acquired at 405 nm using a Multiskan FC ELISA plate reader (Thermo Scientific). Samples were measured at least in duplicates.

For analysis of affinity-maturation (22), results from plates coated with InsA(1) or InsA(4) were calculated by dividing InsA(1) by InsA(4). Here, we used streptavidin with tetra- (ThermoScientific) or monovalent binding capacity. Subsequently, results were stated as relative units [RU].

### Enzyme-Linked Immuno-Spot Assay (ELISpot)

Total splenocytes were measured in triplicates with cell numbers stated in the figure. Particular cell populations were sorted by using a FACS Aria flow cytometer. ELISpot plates were coated with native Insulin (Sigma-Aldrich, Cat. 91077C), NP-BSA (BiosearchTechnologies) anti-IgM (Mabtech). Seeded cells were incubated for 12 – 24 h at 37°C, antigen-specific IgM or IgG was detected *via* anti-IgM-bio and SAV-AP or anti-IgG-bio and SAV-AP (Mabtech). Experiments were done according to the manufacturer’s instructions.

### HEp-2 Slides and Fluorescence Microscopy

HEp-2 slides (EUROIMMUN, F191108VA) were used to asses anti-nuclear-reactivity (ANA) of immunoglobulins from murine sera. Sera of immunized mice on days indicated in the figures or figure legends were diluted to an equal concentration of IgM (approx. 300 ng/mL anti-Insulin-IgM in both immunized samples) and applied onto the HEp-2 slides. Anti-IgM-FITC (eBioscience, Cat. 11-5790-81) was used for visualization of ANA-IgM. A fluorescence microscope Axioskop 2 (Zeiss) and DMi8 software (Leica) were used to analyze stained HEp-2 slides.

### Glucose Level Monitoring

Urine glucose levels were assessed using Combur 10 M Test stripes (Roche Diagnostics, Mannheim). Sterile stripes were used during daily mouse handling and the displayed color after testing was compared to the manufacturer’s standard of glucose levels in mmol/L. An AccuCheck (Roche Diagnostics, Mannheim) blood glucose monitor was used to measure blood glucose levels of mice. Blood was taken freshly from the tail vein from *ad libitum* fed mice and transferred onto sterile test stripes. Glucose levels were measured in mmol/L at days stated in the figures for each mouse per group. Control-immunizations were done with littermates and measured together with experimental mice.

### SDS-PAGE and Coomassie

Samples were separated on 10 – 20% SDS-polyacrylamide gels and incubated with Coomassie (Coomassie brilliant blue R-250, ThermoFisher) for 45 min and subsequently de-stained.

### Pulldown of Total Serum Immunoglobulins

Serum of immunized mice was collected immediately after euthanization. Removal of antigen bound to antibodies was achieved by repeated freeze-thaw cycles of the serum and pH-shift during elution (23). Protein G sepharose beads (ThermoFisher) were used according to the manufacturers protocol and dialyzed overnight in 10 times sample volume in 1x PBS to isolate IgG. For IgM, HiTrap IgM columns (GE Healthcare, Sigma-Aldrich) were used according to the manufacturers protocol and dialyzed overnight in 10 times sample volume 1 x PBS. Quality-control of the isolated immunoglobulins was done *via* SDS-PAGE and Coomassie-staining and the concentration of insulin-specific immunoglobulins determined *via* ELISA.

### Isolation of Insulin-Specific Serum Immunoglobulins

Serum of immunized mice was taken immediately after euthanization and transferred into serum collection tubes. Streptavidin bead columns (Thermo-Scientific, Cat. 21115) were loaded with 10 µg bio-Insulin (BioEagle) to pull down insulin-specific immunoglobulins. The sera were incubated for 90 min at room temperature to ensure sufficient binding of antibodies to the insulin-loaded beads. Purification of the insulin-antibodies was achieved by a pH-shift (pH 2.8) using the manufacturers elution and neutralization solutions. Quality of the isolated immunoglobulins was examined *via* Coomassie-staining and ELISA. For further *in vivo* experiments, the isolated antibodies were dialyzed as described above.

### Bio-Layer Interferometry

Interferometric assays (BLItz device, ForteBio) were used to determine the affinity of protein-protein interactions. Here, we used insulin-specific IgM (see *Isolation of Insulin-Specific Serum Immunoglobulins*) and insulin-bio (ThermoFisher) as a target. The targets were loaded onto Streptavidin biosensors (ForteBio). Binding affinities of IgM to Insulin were acquired in nm. Therefore, the following protocol was used: 30 sec baseline, 30 sec loading, 30 sec baseline, 240 sec association, 60 sec dissociation. For buffering of samples, targets and probes, the manufacturer’s sample buffer (ForteBio) was used.

### Statistical Analysis

Graphs were created and statistical analysis was performed by using GraphPad Prism (version 6.0h) software. The numbers of individual replicates or mice (n) are stated within the figure or figure legends. Data sets were analyzed by D’Agostino & Pearson omnibus normality test and/or Shapiro-Wilk normality test in GraphPad Prism software to determine whether they are normally distributed. If one of the data sets was not normally distributed or the sample number n was too small to perform the normality tests, non-parametric tests were used to calculate p-values. In this study, p-values were calculated by tests stated in the respective figure legends. Students t-tests with Welch’s correction were used to compare two groups within one experiment. P values > 0.05 were considered to be statistically significant (n.s.=not significant; *p < 0.05; **p < 0.01; ***p < 0.001, ****p < 0.0001).

## Results

### The Presence of Soluble Antigen Suppresses IgG Production

A main conclusion of the adaptive tolerance and responsiveness model is that the ratio of IgM to IgD on mature B cells together with the ratio of soluble (monovalent) to complex (polyvalent) antigen in the microenvironment determine the threshold for B cell activation and antibody secretion. This proposes a dynamic model, in which the activation of mature B cells is not an unavoidable step after antigen-binding, as activation is regulated by the class of BCR expressed and the ratio of antigen-valences. Consequently, autoreactive mature B cells can exist without inducing destructive autoantibody responses as long as the cognate autoantigen is monovalent.

**Figure 1:**
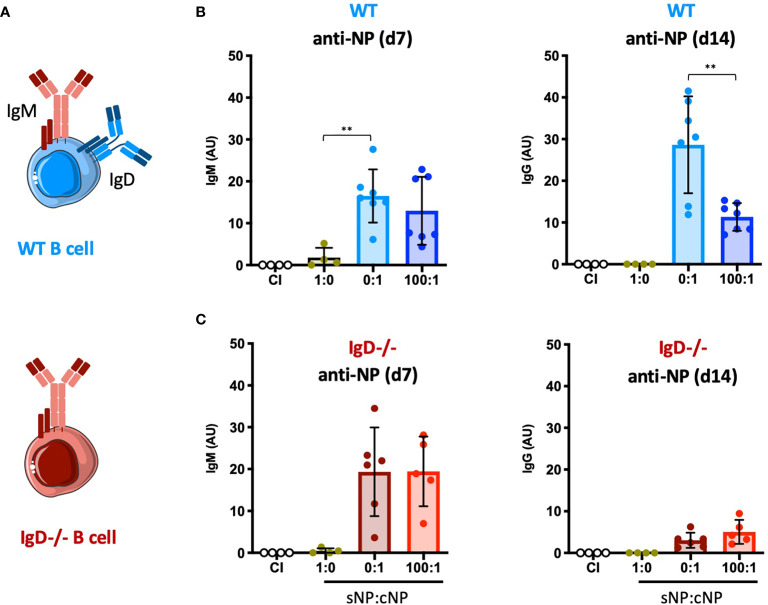
Modulation of immune responses by monovalent antigen requires expression of the IgD-class BCR. **(A)** Schematic illustrations of a WT and IgD-deficient B cell. **(B, C)** NP-reactive serum immunoglobulins of WT mice **(B)** and IgD-deficient mice **(C)** injected with PBS (control immunization (CI), n=4/group), 4-hydroxy-3-nitrophenylacetyl only (sNP: 1:0; WT n=4, IgD-deficient n=4), complex NP (cNP: 0:1; WT n=7, IgD-deficient n=6) and soluble:complex NP (100:1; WT n=7, IgD-deficient n=5). Left panel showing anti-NP IgM on day 7, right panel shows anti-NP-IgG on day 14. Mean ± SD, students t-tests with Welch’s correction were used to compare two groups within one experiment. **P < 0.01.

To test the concept of adaptive tolerance and responsiveness of B cells *in vivo*, we performed immunization experiments using the hapten NP (4-hydroxy-3-nitrophenylacetyl) coupled to KLH (Keyhole Limpet Hemocyanin) as a carrier (**Supplementary Figure 1A**). To this end, groups of wild-type (WT) mice were injected with either NP as soluble compound (sNP) or NP-KLH, referred to as polyvalent complex antigen (cNP), at similar molarity for NP (NP(30)KLH). NP-specific antibody responses were determined at day 7 (IgM) and day 14 (IgG) post immunization (**Figures 1A, B**). Similar to control immunization (CI) containing only CpG but no antigen, injection of only soluble hapten (sNP:cNP, 1:0) failed to induce clear IgM or IgG antibody responses, while injection of cNP (sNP:cNP, 0:1) was able to induce both. Adding sNP to cNP at 100:1 ratio did not alter anti-NP-IgM antibody responses. However, the anti-NP-IgG response was significantly impeded at 100:1 ratio for sNP to cNP (**Figure 1B** and **Supplementary Figure 1D**). Importantly, the IgG response to the carrier (KLH) was comparable regardless of the amount of soluble hapten added (**Supplementary Figure 1B**).

To further confirm these findings, we performed ELISpot assays for assessing the number of NP-specific antibody secreting cells. In agreement with the serum immunoglobulin data, the ELISpot results showed that adding sNP to cNP at 100:1 ratio reduces the number of IgG secreting cells whereas IgM secreting cells were unaffected (**Supplementary Figure 1C**). These data support the concept of soluble monovalent antigen interfering in immune responses to complex forms of the same antigen.

A crucial pillar of the concept proposes that the presence of IgD-class BCR is important for the observed regulation (**Figures 1A–C**). Thus, we tested the role of IgD by conducting the NP immunization experiments in IgD knockout mice lacking IgD-class BCR (**Figure 1A**). Notably, expression levels of surface IgM on marginal zone and follicular B cells of IgD-deficient (IgD-/-) mice were comparable to WT mice (**Supplementary Figure 2**).

IgD-deficient mice showed slightly elevated IgM responses as compared to WT mice. However, the IgG response was weaker and no inhibitory effect on IgG was observed when sNP was added to cNP prior to immunizing IgD-deficient mice (**Figure 1C** and **Supplementary Figure 1D**).

Together, these data suggest that mature B cells are able to fine-tune their immune response according to the antigen-valency, thereby leading to distinct IgM and IgG responses to different epitopes of the same antigen.

### Accelerated Primary Immune Response in IgD-Deficient Mice

**Figure 2:**
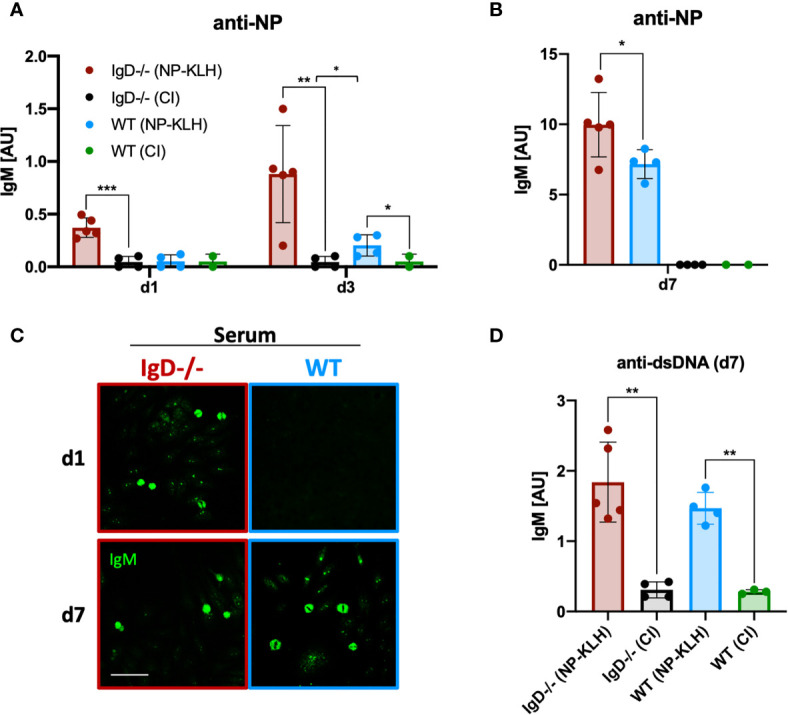
IgD-deficient mice mount robust polyreactive IgM responses one day after immunization. **(A, B)** Serum immunoglobulin titers of NP-KLH (IgD-deficient n=5, WT n=4) and CpG ODN1826 (control immunization: CI, IgD-deficient n=4, WT n=2) injected mice measured by ELISA. NP-reactive IgM of day 1 and 3 **(A)** and day 7 **(B)**. Mean, ± SD, statistical significance was calculated by using Kruskal-Wallis-test. **(C)** Serum immunoglobulins reactive to self-molecules (DNA/RNA) of NP-KLH and CI injected IgD-deficient and WT mice tested *via* HEp2 slides. Fluorescence microscopy images are representative for three independent experiments. Scale bar: 10 µm. **(D)** Serum immunoglobulin titers of NP-KLH (IgD-deficient n=5, WT n=4) and CI injected (IgD-deficient n=4, WT n=2) mice measured by ELISA. dsDNA-reactive IgM of day 7 post immunization. Students t-tests with Welch’s correction were used to compare two groups within one experiment. Mean, ± SD. *P < 0.05, **P < 0.01.

To further investigate the dynamics of immune responses in IgD-deficient mice, we monitored early antibody production. Compared to WT mice, IgD-deficient mice showed a NP-reactive IgM response already at day 1 after immunization (**Figure 2A**). This response was further amplified at day 3 and peaked at day 7 (**Figure 2B**). In contrast, no antibody response was observed at day 1 in WT mice. By day 3, WT mice showed a slight NP-reactive IgM response which peaked at day 7, however, remained slightly weaker than the day 7 NP-reactive IgM response in IgD-deficient mice (**Figures 1A, B**). To further characterize the specificity of the induced antibody response we performed classical autoreactivity assays including indirect immunofluorescence (IF) on HEp-2 slides and ELISA for anti-double stranded DNA (dsDNA). These experiments show that primary IgM antibodies, detected at day 1 throughout day 7 of IgD-deficient mice or by day 7 of WT mice, are autoreactive determined by recognition of nuclear structures (**Figures 2C, D**). Importantly, the control immunizations using only the adjuvant CpG showed no induction of anti-dsDNA antibodies as compared with unimmunized mice (data not shown). Further, to exclude a role of CpG in the production of autoreactive IgM, we stained HEp-2 slides with sera of control immunizations. Neither IgD-deficient mice nor WT mice showed elevated levels of autoreactive IgM in control immunizations with PBS or CpG alone (**Supplementary Figure 3**).

In summary, these results show that primary IgM responses are autoreactive and that IgD-deficiency allows rapid primary immune responses.

### Sustained Primary Immune Response to Autoantigen in IgD-Deficient Mice

After testing hapten-specific antibody responses, we investigated whether the production of autoreactive primary antibodies in IgD-deficient mice differ when using autoantigens. To avoid the usage of transgenic mice that artificially harbor mono-specific B cells expressing a defined BCR that recognizes either a transgene product or endogenous structure, we selected insulin-related autoantigens as a physiologically relevant system for autoimmune diseases (Amendt and Jumaa 2021, *in press*).

**Figure 3:**
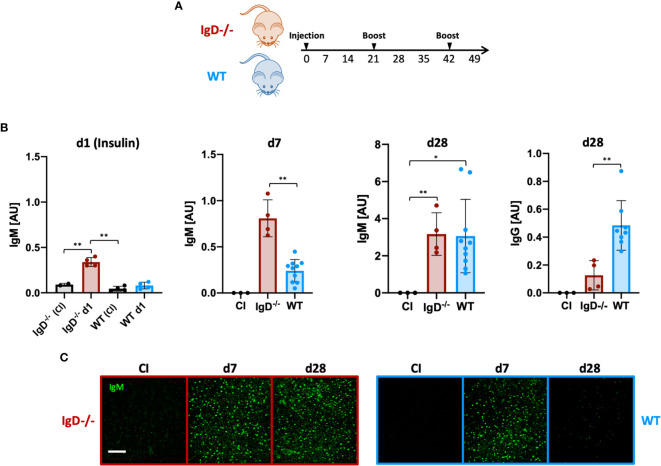
The IgD-class BCR is required to prevent rapid immune response to autoantigens and induces affinity maturation. **(A)** Schematic immunization schedule of IgD-deficient and WT mice injected with InsA-KLH + CpG ODN1826 on day 0 and InsA-KLH on days 21 and 42. **(B)** Serum immunoglobulin titers reactive to native insulin of IgD-deficient (n=4) and WT (n=10) mice immunized with InsA-KLH or CI (n=3) measured by ELISA. Days are indicated in the figure. Mean, ± SD. Students t-tests with Welch’s correction were used to compare two groups within one experiment. **(C)** Serum immunoglobulins reactive to self-molecules (DNA/RNA) of InsA-KLH and CI injected IgD-deficient (n=5/day) and WT (n=5/day) mice tested *via* HEp2 slides. Fluorescence microscopy images are representative of three independent experiments. Scale bar: 10 µm *P < 0.05, **P < 0.01.

To this end, we performed immunization experiments using an Insulin-A chain-derived peptide, referred to as InsA which is the most abundant epitope in autoantibody responses against insulin (24). The selected peptide was covalently coupled to the carrier KLH to generate a complex polyvalent antigen (InsA-KLH) which was then used in immunization experiments (**Figure 3A**). Subsequently, we monitored the antibody responses against the InsA peptide and native insulin to confirm the induction of harmful autoantibody responses. We found that InsA-KLH induced IgM antibody responses recognizing native insulin already at day 1 after immunization (**Figure 3B**). WT mice showed no insulin-reactive IgM at day 1. By day 7, WT mice showed anti-insulin IgM which, however, was reduced as compared to IgD-deficient mice (**Figure 3B**). By day 28 comparable amounts of insulin-reactive IgM were detected in both IgD-deficient and WT mice. However, only WT mice showed a considerable increase of insulin-specific IgG (**Figure 3B**, right panel). This IgG is responsible for the elevated blood glucose levels detected in these mice (see below).

Importantly, ELISpot analysis confirmed the increased number of anti-insulin antibody secreting cells in IgD-deficient mice at day 1 after immunization (**Supplementary Figure 4**).

To evaluate the polyreactive potential of the elicited anti-insulin IgM, we performed HEp-2 slides. The data show that anti-insulin IgM remained polyreactive throughout day 28 (boost on day 21), whereas the anti-insulin IgM of WT mice was no longer polyreactive (**Figure 3C**). Importantly, immunization with mixtures of InsA-KLH and soluble InsA peptide at 1:100, respectively, resulted in highly elevated blood glucose concentrations at day 1 and were therefore discontinued (see below, data not shown).

Together, our data suggest that IgD-deficient B cells elicit a rapid and strong primary immune response that persists for longer periods compared to WT mice. Thus, the shift into secondary, mature immune responses is delayed in IgD-deficient mice. Interestingly, IgD, which is a marker for mature B cells, is required for the transition of the immune response from primary to secondary phases.

### Delayed Affinity Maturation Is Associated With Sustained Autoimmunity in IgD-Deficient Mice

**Figure 4:**
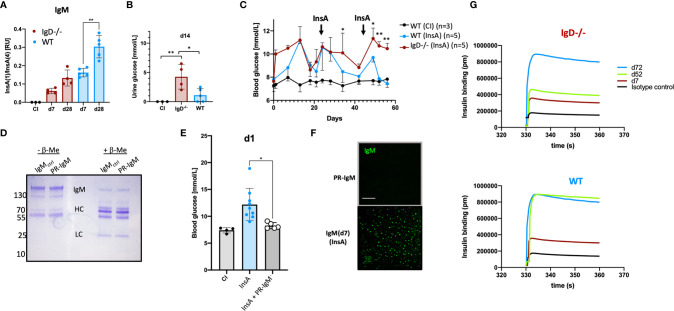
The IgD-class BCR is required for affinity maturation of insulin-IgM to be protective and prevent autoimmune pathology. **(A)** Affinity of IgM to InsA peptides of InsA (InsA-KLH + CpG ODN1826) immunized IgD-deficient (n=4), WT (n=5) and CI (n=3) mice measured by peptide-ratio ELISA (22).Plates were coated with Streptavidin bearing one [InsA(1)] or four [InsA(4)] biotin binding sites. Mean, ± SD, statistical significance was calculated by using repeated measure ANOVA-test. **(B)** Urine glucose values (mmol/L) measured by commercial urine stripes (Roche) of IgD-deficient (n=4) and WT (n=5) mice immunized with InsA-KLH or CI (n=3). Mean, ± SD, statistical significance was calculated by using Kruskal-Wallis-test. **(C)** Blood glucose values (mmol/L) of IgD-deficient (n=4) and WT (n=5) mice immunized with InsA. Injections were done on day 0 (InsA-KLH + CpG ODN1826), day 21 (InsA-KLH), day 42 (InsA-KLH). Mean, ± SD, statistical significance was calculated by using repeated measure ANOVA-test comparing red and blue graphs. **(D)** Coomassie-stained SDS-PAGE showing reduced (+ β-ME) and non-reduced (- β-ME) IgM of cInsA and control immunized mice. Total serum IgM was isolated *via* HiTrap IgM columns (InsA d85 refers to PR-IgM). IgM monomer: 150 kD, IgM HC: 70 kD, IgM LC: 25 kD. **(E)** Blood glucose values (mmol/L) of IgD-deficient immunized with InsA-KLH (n=9), WT control immunized mice (n=4) and IgD-deficient mice immunized with InsA-KLH and injected with PR-IgM i.v. (n=5). Mean, ± SD, statistical significance was calculated by using Kruskal-Wallis-test. **(F)** Serum immunoglobulins reactive to self-molecules (DNA/RNA) of IgM (PR-IgM and day 7 primary IgM) isolated from InsA-KLH immunized mice tested *via* HEp2 slides. Fluorescence microscopy images are representative for three independent experiments. Scale bar: 10 µm. **(G)** Interferometric assay to determine the affinity of IgM to insulin. Insulin-specific isolated IgM of IgD-deficient (top panel) or WT (bottom panel) mice immunized with InsA. Affinity of IgM of different days is shown in pm. Graphs are representative of three independent experiments *P < 0.05, **P < 0.01.

Affinity maturation of antibody responses was reported to be delayed in IgD-deficient mice (14). To test this for self-molecules, we performed ELISA to determine the binding efficiency of the insulin-specific antibodies to high-valence or low-valence antigen. To this end, we used monovalent and polyvalent streptavidin and biotinylated InsA peptides to generate monovalent InsA(1) and polyvalent InsA(4) streptavidin complexes. Similar to previous reports (14, 22) an increased ratio of InsA(1)/InsA(4) indicates increased affinity. Our experiments revealed no significant increase of the affinity of insulin-reactive IgM antibodies between primary (d7) and secondary (d28) immunization for IgD-deficient mice. However, insulin-reactive IgM of WT mice showed a clear increase in InsA(1)/InsA(4) ratio which is characteristic for affinity maturation (**Figure 4A**). We confirmed these results by examining the direct binding affinity of insulin-specific IgM of different days from WT and IgD-deficient mice. Interferometric assays revealed that WT mice already reach high affinity insulin-IgM at day 52, whereas IgD-deficient mice do not reach a comparable level of affinity before day 72 (**Figure 4G**). Interestingly, both IgD-deficient mice and WT mice showed clear signs of diabetes as detected by glucose amount in the urine and increased blood glucose levels. However, the symptoms of diabetes were more severe and evident already at day 1 in IgD-deficient mice as compared to WT mice (**Figures 4B, C**). Importantly, the increase in blood glucose concentrations in WT mice gradually declined after repeated boost immunizations and shortly after the second boost at day 42 the WT mice became resistant to InsA-induced diabetes (**Figure 4C**). IgD-deficient mice in contrast showed sustained diabetes symptoms throughout day 60 after immunization (**Figure 4C**). In fact, IgD-deficient mice required a third boost by day 70 to develop resistance to InsA-induced diabetes (**Supplementary Figures 5A, B**).

To show that high-affinity IgM generated during secondary booster immunizations is responsible for the control of diabetes induced by InsA immunization, we isolated insulin-specific IgM from WT mice after secondary immunization and injected it into IgD-deficient mice shortly after immunization with InsA-KLH. The results show that the isolated high-affinity IgM protected the IgD-deficient mice from developing diabetes at day 1 and therefore we refer to this IgM as protective IgM (PR-IgM) (**Figures 4D, E**). The underlying mechanism of PR-IgM generation and characterization is part of Amendt and Jumaa 2021 (*in press*). Similar to the above-mentioned results (**Figure 3D**) we performed indirect IF to confirm that affinity maturation resulted in PR-IgM which is highly specific for insulin without binding other autoantigens (**Figure 4F**).

These data show that defective affinity maturation in IgD-deficient mice (**Supplementary Figure 6**) leads to insufficient production of protective highly specific IgM thereby resulting in sustained autoimmune disorder.

### Rapid Activation of IgD-Deficient B Cells After Immunization

**Figure 5:**
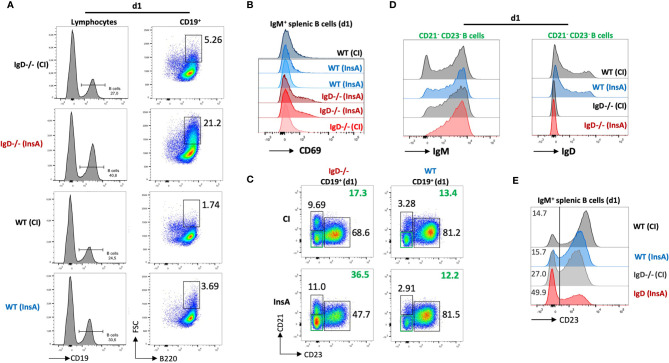
The IgD-class BCR controls a rapidly responding CD21^-^CD23^-^ B cell population that is able to secrete autoantibodies 24 hours after immunization. **(A – E)** Flow cytometric analysis of IgD-deficient (n=4/group) and WT (n=4/group) mice immunized with InsA-KLH + CpG ODN1826 or CpG ODN1826 (CI). All panels show representative plots pre-gated on lymphocytes (FSC/SSC), single cells (SSC-H/SSC-W) and viable cells (fixable viability dye, FVD^-^). **(A)** Histogram of CD19 expression used to gate B cells (CD19^+^) within lymphocyte gate (left). Enlarged (activated) IgM^+^ B cells (FSC^hi^ B220^+^) within B cell gate (right). **(B)** Histogram showing activated B cells by CD69 expression pre-gated on IgM^+^ B cells. **(C)** Representative plot showing marginal zone B cells (CD21^hi^ CD23^lo^), follicular B cells (CD21^lo^ CD23^hi^) and CD21^-^ CD23^-^ (double negative) B cell population in InsA and CI immunized IgD-deficient or WT mice. **(D)** Histograms of CD21^-^CD23^-^ B cells IgM expression (left) and IgD expression (right). **(E)** Histogram showing CD23 expression in IgM^+^ splenic B cells.

To examine early changes on B cells after immunization, we performed FACS experiments to analyze lymphoid organs after immunization with InsA-KLH. This analysis revealed that immunization induced a substantial expansion of splenic B cells in IgD-deficient mice as compared to WT mice at day 1 (**Figure 5A**). Notably, the expansion of splenic B cells in IgD-deficient mice was associated with increased cell size, as shown by forward-scatter (FSC), in the IgD-deficient mice (**Figure 5A**). In full agreement, a considerable fraction (>21%) of B cells expressed the activation marker CD69 after immunization of IgD-deficient mice with InsA-KLH. Similar to control immunization of IgD-deficient mice with CpG alone, WT mice immunized with InsA-KLH or CpG alone showed a limited fraction (about 2-5%) of CD69 expressing cells (**Figure 5B**).

Further characterization revealed that a population of CD23-/CD21- cells was increased in IgD-deficient mice immunized with InsA-KLH compared to immunized WT counterpart or IgD-deficient mice from control immunization (**Figure 5C**). The CD23-/CD21-cells correspond to activated B cells (**Supplementary Figures 7A–C**) which predominantly express IgM-class BCR and intermediate amounts of IgD in WT mice (**Figure 5D**). In particular, CD23 expression on B cells is greatly down-regulated in InsA-KLH immunized IgD-deficient mice on day 1 as compared to controls (**Figure 5E**). This is consistent with available data showing that CD23 is downregulated upon B cell activation (25).

Thus, our results suggest that IgD-deficient B cells are rapidly activated after immunization and that the responsive cells are CD23-/CD21- with elevated levels of IgM BCR expression.

### Antibody Secretion by CD23-/CD21- Cells

**Figure 6:**
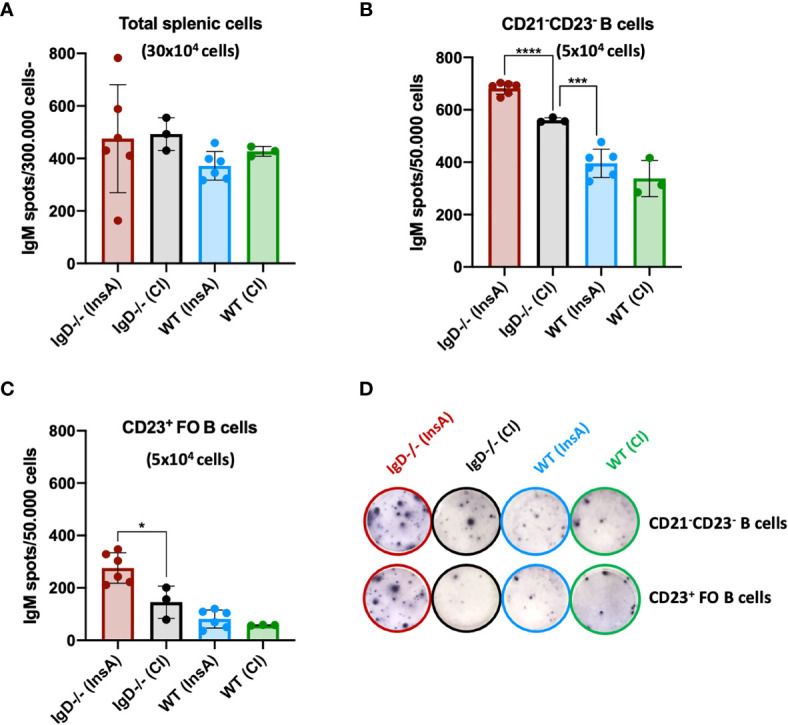
The CD21^-^ CD23^-^ B cell population is the major source of IgM-secreting cells under the control of the IgD-class BCR. **(A–D)** ELISpot analyses showing IgM secreting splenic cells of InsA-KLH + CpG ODN1826 or control (CpG ODN1826) immunized (CI) mice 24 hours after injection. IgM secretion by **(A)** total splenic cells, **(B)** CD21^-^CD23^-^ sorted B cells, **(C)** CD23^+^ follicular B cells, **(D)** representative images of ELISpot wells of indicated cell numbers, genotypes and immunizations. Two independent experiments with n=3/group for CI and n=6/group for InsA-KLH were performed. Mean, ± SD, students t-tests with Welch’s correction was used to compare two groups within one experiment. *P < 0.05, ***P < 0.001, ****P < 0.0001.

Our recent data showed that CD23-/CD21- cells are antibody-secreting cells. Therefore, we investigated antibody secretion by splenocytes at day 1 after immunization. ELISpot analysis showed that the proportion of antibody-secreting cells is slightly increased in IgD-deficient mice when total splenic cells were used (**Figure 6A**). However, when ELISpot analysis was performed after FACS sorting for CD23-/CD21- cells or CD23+ follicular B cells, we found that antibody secretion was predominantly associated with CD23-/CD21- cells (**Figure 6B**). The increased proportion of the CD23-/CD21- in InsA-KLH immunized IgD-deficient mice was associated with increased proportion of antibody-secreting cells (**Figure 6B**). Interestingly, only a small fraction of follicular CD23+ B cells from WT mice developed into antibody-secreting cells and no effect of the immunization was observed at day 1 while IgD-deficient follicular CD23+ B cells showed an increase in antibody-secreting cells (**Figures 6C, D**). In order to further characterize the CD23-/CD21- B cell population, we performed FACS experiments. We found that these cells lack the typical markers for plasma cells (CD138) or B-1 B cells (CD43, CD11b). Interestingly, however, these cells showed highly increased MHC-class II expression suggesting that they are activated B cells (**Supplementary Figure 8**).

Interestingly, IgD-deficient B cells in peritoneal cavity further showed an increase of CD138^+^ cells at day 1 after immunization, whereas WT counterparts remained unchanged (**Supplementary Figure 9**).

In summary, these data suggest that immunization results in increased CD23-/CD21- antibody-secreting cells that develop within one day in IgD-deficient mice.

### Primary Immune Responses Have Restricted Polyreactivity

**Figure 7:**
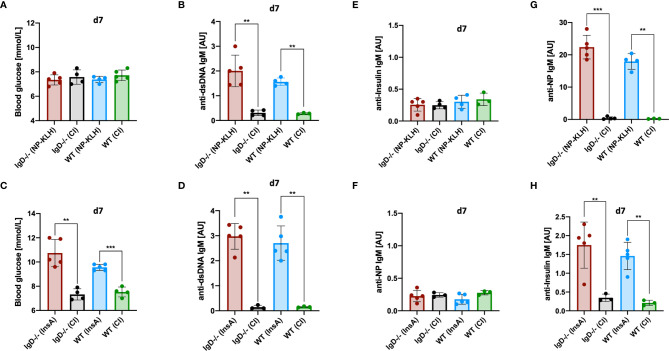
Primary IgM is antigen-specific and polyreactive but not cross-reactive. **(A, C)** Blood glucose levels (mmol/L) of IgD-deficient and WT mice immunized with NP-KLH + CpG and controls (CpG-ODN1826) **(A)**, or immunized with InsA-KLH + CpG and controls **(C)**. Mean, ± SD, statistical analysis was done by using Kruskal-Wallis-test. **(B, D)** Serum immunoglobulin titers of IgD-deficient and WT mice immunized with either NP-KLH + CpG and controls **(B)** or InsA-KLH + CpG and controls **(D)** reactive to dsDNA measured by ELISA. Mean, ± SD, statistical analysis was done by using Kruskal-Wallis-test. **(E–H)** Serum immunoglobulin titers of IgD-deficient and WT mice immunized with either NP-KLH + CpG or InsA-KLH + CpG and controls reactive to Insulin (top panel) or InsA-KLH + CpG and controls immunized mice reactive to NP (bottom) **(E, F)** and reactive to NP or Insulin **(G, H)** measured by ELISA. Mean, ± SD, statistical analysis was done by using Kruskal-Wallis-test **P < 0.01, ***P < 0.001.

The results presented above indicate that primary immune responses are autoreactive regardless of the utilized antigen. In fact, primary anti-NP- as well as primary anti-insulin-IgM showed nuclear staining in HEp-2 slides indicative of polyreactive behavior. In full agreement, the primary anti-NP-IgM also showed anti-dsDNA binding in ELISA experiments (**Figure 2D**). Subsequently, we tested the hypothesis whether primary IgM immune responses might always induce the same class of autoreactive B cells that might be omnipotent with regard to autoantigen binding. To this end, we tested whether the anti-NP primary immune response induces diabetes symptoms due to binding to insulin. However, despite the increased polyreactivity, neither IgD-deficient nor WT mice showed any changes in blood glucose levels in the course of NP immunization (**Figures 7A, C**). Further, InsA-KLH immunized WT and IgD-deficient mice showed an expected increase in blood glucose levels (**Figure 7C**). The primary IgM of these mice also showed significant polyreactivity, as determined by anti-dsDNA ELISA (**Figures 7B, D**). Nevertheless, no increased anti-insulin binding was observed in the sera of IgD-deficient or WT mice after NP immunization (**Figures 7E, G**, top). Vice versa, InsA-KLH immunized mice did not show increased NP binding (**Figures 7F, H**, bottom).

Together, these data suggest that, despite their increased autoreactivity to nuclear structures and dsDNA, primary IgM immune responses in IgD-deficient as well as in WT mice have no infinite polyreactivity. In conclusion, primary IgM responses appear to be broadly poly- and self-reactive, but still remain somehow specific for their cognate antigen.

## Discussion

In this study we investigated the importance of the IgD-class BCR expression for mature B cell function *in vivo*. Notably, the flexible hinge region in the HC enables IgD expressing B cells to distinguish between polyvalent complex form and monovalent soluble form of the same antigen (10, 11). This ability of IgD results in an unique control mechanism for B cell activation, in which the ratio of polyvalent to monovalent antigen determines B cell responsiveness. In fact, as monitored by calcium mobilization *in vitro*, polyvalent antigen activates signaling in IgD expressing cells while monovalent antigen interferes with this responsiveness (10, 11, 14, 19, 26).

To investigate the importance of the unique IgD function *in vivo*, we determined the antibody immune response to monovalent or polyvalent forms of an hapten or a disease-relevant antigen (insulin) in wildtype as compared to IgD-deficient mice (10, 12, 27).

The results show that monovalent antigen suppresses IgG antibody production triggered by cognate polyvalent antigen in IgD expressing cells regardless of whether the antigen belongs to self or non-self. In the absence of IgD, soluble antigen cannot alter the IgG antibody response while an immediate production of primary antigen-reactive IgM antibody response is triggered and maintained for extended times as compared with IgD expressing cells in WT mice. This phenomenon might have crucial consequences for IgG antibody responses against autoantigens which could be present in complexes as a result of cell death, protein turnover or interaction with foreign structures. Since autoreactive IgG is a hallmark of autoimmune diseases, our data suggest the presence of IgD together with an excess of soluble monovalent form of the same antigen interferes with the activation of autoreactive IgG antibody secretion. Because the antibody secreting cells described in this study lack typical B1-B cell markers, the contribution of B1-B cells to the deregulation of primary responses in IgD-deficient mice is unclear. However, the reduced IgD-class BCR expression detected on B1-B cells suggests that these cells are prone to the induction of rapid primary immune responses targeting self and non-self which is in agreement with available data (28–30).

Interestingly, IgD expression is also required for efficient activation of memory responses as indicated by the decreased IgG responses to NP and InsA in IgD-deficient mice compared to wildtype controls. Thus, IgD is required for both triggering an efficient memory immune response and at the same time for avoiding such immune responses if the activating antigen is self, which is distinguished as mainly monovalent non-complex antigen. Given that the generation of long-lasting antibody memory responses, which are not self-destructive, is essential for effective immune protection and that IgD is required for these processes, it can be concluded that IgD is a key regulator for balanced antibody responses. This is in full agreement with previous results suggesting that IgD is required for acceleration of affinity maturation in early phases of the immune response (14). Moreover, the suggested role of IgD in affinity maturation corresponds to the prolonged phase of primary IgM antibody response in IgD-deficient mice. Remarkably, this primary IgM is autoreactive and might lead to prolonged autoimmune damage as in the case of insulin-derived peptides. Thus, another important characteristic of IgD function is limiting the duration of autoreactive primary IgM responses by accelerating the generation of highly specific IgM that protects autoantigen from autoimmune destruction. In fact, the generation of protective memory IgM is delayed in the IgD-deficient mice.

Interestingly, IgD has been associated with the control of autoimmunity and quiescence of autoreactive B cells, as IgD BCR was suggested to be less sensitive than IgM BCR to endogenous antigen (31, 32). However, the underlying mechanism for this control of autoreactive B cells remained unclear.

Our results suggest that IgD BCR regulates the responsiveness and antibody secretion *in vivo* by sensing antigen-valency regardless of whether the antigen belongs to self or non-self. An excess of monovalent antigen relative to polyvalent antigen interferes with IgG production and activates the generation of high affinity IgM antibodies by triggering IgD expressing mature B cells. An excess of polyvalent antigen leads to efficient IgG memory responses by activated mature B cells. The result is fine-tuned sensing of antigen forms and consequent regulation of the dynamics of B cell activation and antibody responses. This is defective in IgD-deficient B cells which are immediately activated already at day 1 after immunization suggesting that the presence of IgD prevents deregulated activation of B cells. This is in agreement with recent data showing that the IgD-class BCR is required for maintaining the organization of signaling complexes in distinct nanoclusters at the cell surface (21, 33). This organization keeps naïve B cells in a resting state, in which stimulatory co-receptors are confined in the IgD BCR nanoclusters and thus away from IgM BCR, which seems to trigger robust cell activation when associated with the stimulatory molecules. If this organization is disrupted, which usually occurs upon antigen binding, the stimulatory co-receptors traffic into the IgM nanoclusters resulting in B cell activation (21, 33). It is likely that IgD-deficient B cells have disrupted organization of nanoclusters as stimulatory co-receptors cannot be confined to IgD nanoclusters and may thus easily associate with IgM resulting in accelerated activation of IgD-deficient B cells. It was recently reported that IgM and IgD-class BCR play opposing roles in B cell survival and that the IgD-class BCR negatively affects B cell activation and differentiation (34). Here, we show that the IgD-class BCR prevents rapid B cell activation and IgM antibody secretion, which is in agreement with the notion that IgD is likely a negative regulator of B cell activation. Furthermore, available data suggest an important role of IgD for CXCR4 function, a chemokine receptor required for germinal center function and thus affinity maturation (35). These data support the uniqueness of the IgD-class BCR and underline the importance of nanocluster organization for the negative regulatory role of IgD (35). In fact, IgD-deficient B cells might have a lower threshold for activation as the confinement in the regulatory IgD clusters is blocked. This is in agreement with available data suggesting that IgM BCR, but not IgD, is required for the survival of transformed B cells (36).

Other studies proposed that the IgM-class BCR induces functional unresponsiveness (so-called anergy), while the IgD-class BCR attenuates this anergy reaction (37). This anergy reaction was associated with the induction of transcription factors such as EGR1/EGR2 (37). However, the induction of EGR1/EGR2 was assigned to anergy and not to IgD expression. Moreover, the IgD-deficient B cells expressing only IgM were proposed to be prone to rapid anergy when encountering self-antigen, as the lack of IgD-class BCR prevents the proposed attenuation of anergy and leads to irreversible unresponsiveness. Consequently, IgD-deficient mice should not be able to mount any autoantibody responses. In contrast, our study shows that the absence of the IgD-class BCR leads to rapid and uncontrolled autoantibody responses when encountering autoantigen complexes suggesting a repressive role for IgD.

Importantly, we have recently shown that the responsiveness of B cells is regulated by unique features of the IgD-class BCR being responsive to polyvalent antigen while being regulated by monovalent antigen (10, 11).

In addition to the role of IgD in controlling B cell activation and proliferation, cells lacking IgD can produce primary IgM but are defective in the generation of protective high affinity IgM. This defect might lead to the production of autoreactive IgG and autoimmune diseases such as systemic lupus erythematosus (SLE) (38–41), Sjögren syndrome (39, 42–44) or inflammatory myopathy (45, 46) which are characterized by autoreactive IgG antibodies recognizing DNA or nuclear structures. The defective generation of high affinity protective IgM by IgD-deficient cells is in agreement with previous data showing that mice deficient for secreted IgM (sIgM) are prone to autoimmunity (47–50). Notably, sIgM-deficient mice developed high titers of anti-dsDNA-IgG in young age (47–50).

In contrast to IgG, IgM expressing B cells recognizing DNA or nuclear structures seem to be abundant in the naïve B cell repertoire resulting in primary responses that are polyreactive. Remarkably, this polyreactivity is partial as both antigens used in this study, NP-KLH and InsA-KLH, led to primary IgM reactive to dsDNA and nuclear structures but lacked reactivity to the respective other antigen. It is conceivable that the partial polyreactivity of primary IgM antibodies is not random and is rather required as part of an efficient primary immune response, in which reactivity with common autoantigens facilitates the generation of immune complexes that are required for the activation of efficient secondary antibody responses. However, a defect in the production of protective IgM or a deregulated switch to IgG can convert the important property of complex formation into an autoimmune disease. Prior studies using pentameric IgM Fc-receptor (FCMR) deficient mice (51, 52) further underline the importance of IgM in initiating powerful secondary responses. In particular, these mice show increased formation of germinal centers, increased plasma cell differentiation and increased autoantibody production, which is in line with the proposed role for primary IgM in our study (51–53).

In summary, our findings suggest that IgD is required for controlling the duration of primary autoreactive IgM production and to control the maturation of secondary antibody responses. For foreign antigens, this maturation results in efficient generation of IgG memory responses. For autoantigens, IgD-mediated maturation of antibody responses leads to efficient generation of high affinity IgM autoantibodies that protect autoantigens. Thus, defective IgD function results in deregulated activation of B cells and may well lead to defective immune responses, autoimmune diseases or uncontrolled proliferation.

## Data Availability Statement

The original contributions presented in the study are included in the article/**Supplementary Material**. Further inquiries can be directed to the corresponding author.

## Ethics Statement

The animal study was reviewed and approved by Regierungspräsidium Tübingen (license 1484), Baden-Württemberg, Germany.

## Author Contributions

TA and OA planned, performed and analyzed experiments, interpreted data, and contributed to writing of the manuscript. AL performed and analyzed FACS experiments. GA, MY, and CS performed ELISA experiments. HJ designed the experiments, wrote the manuscript, and supervised the study. All authors contributed to the article and approved the submitted version.

## Funding

This work was supported by the DFG through TRR130 (B cells and beyond) project 01, SFB1074 (Experimental Models and Clinical Translation in Leukemia), SFB 1279 (Exploration of the Human Peptidome), and ERC advanced grant (694992), DFG single grant, JU 463/5-1.

## Conflict of Interest

The authors declare that the research was conducted in the absence of any commercial or financial relationships that could be construed as a potential conflict of interest.

## Publisher’s Note

All claims expressed in this article are solely those of the authors and do not necessarily represent those of their affiliated organizations, or those of the publisher, the editors and the reviewers. Any product that may be evaluated in this article, or claim that may be made by its manufacturer, is not guaranteed or endorsed by the publisher.
