# Supplementary figures and images for: Primary Immune Responses and Affinity Maturation Are Controlled by IgD
Source: Front Immunol. 2021 Aug 9;12:709240. doi: 10.3389/fimmu.2021.709240 (PMC8381280; doi:10.3389/fimmu.2021.709240)

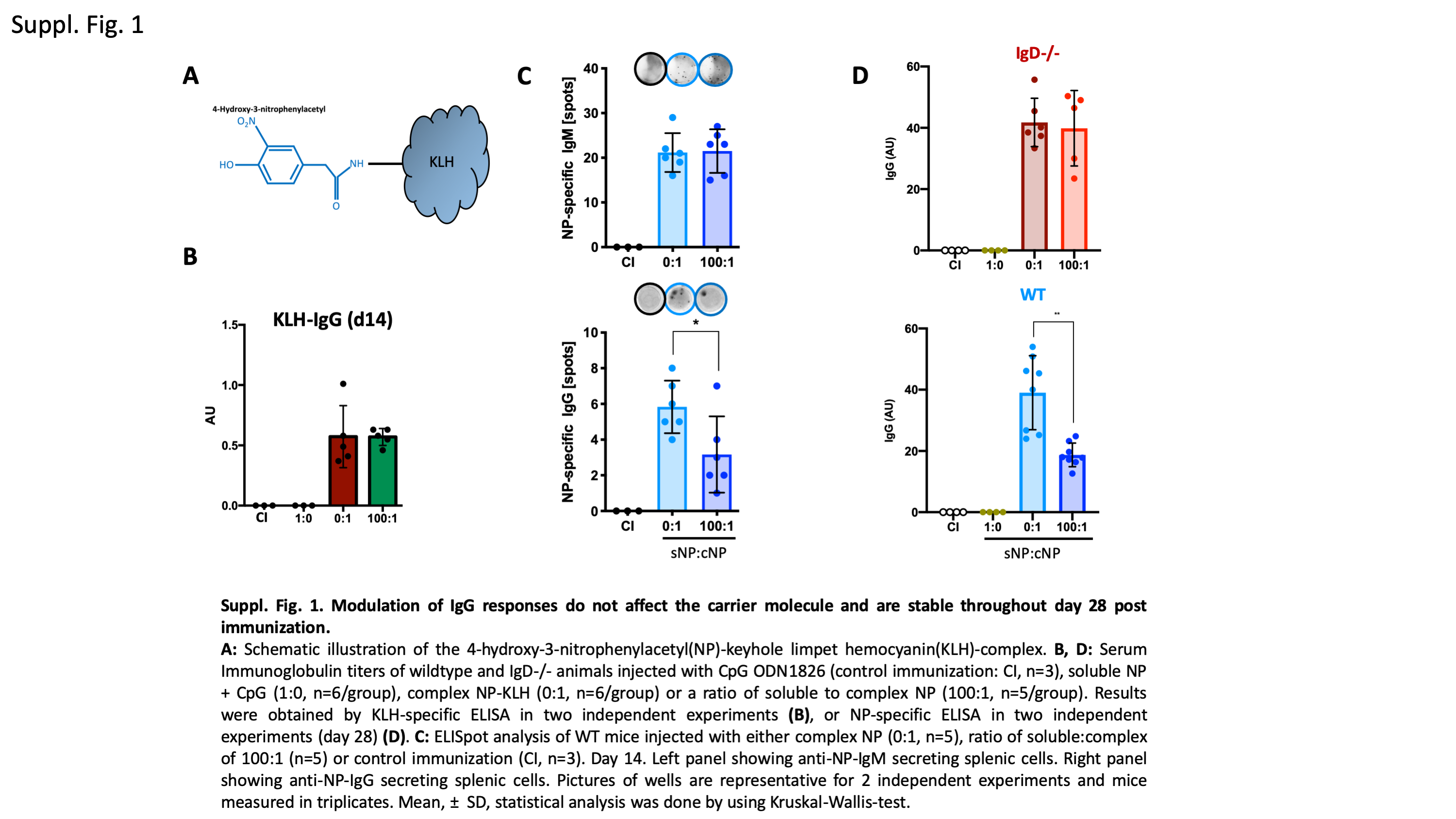

Supplement: Supplementary file 1 [file Image_1.tiff]

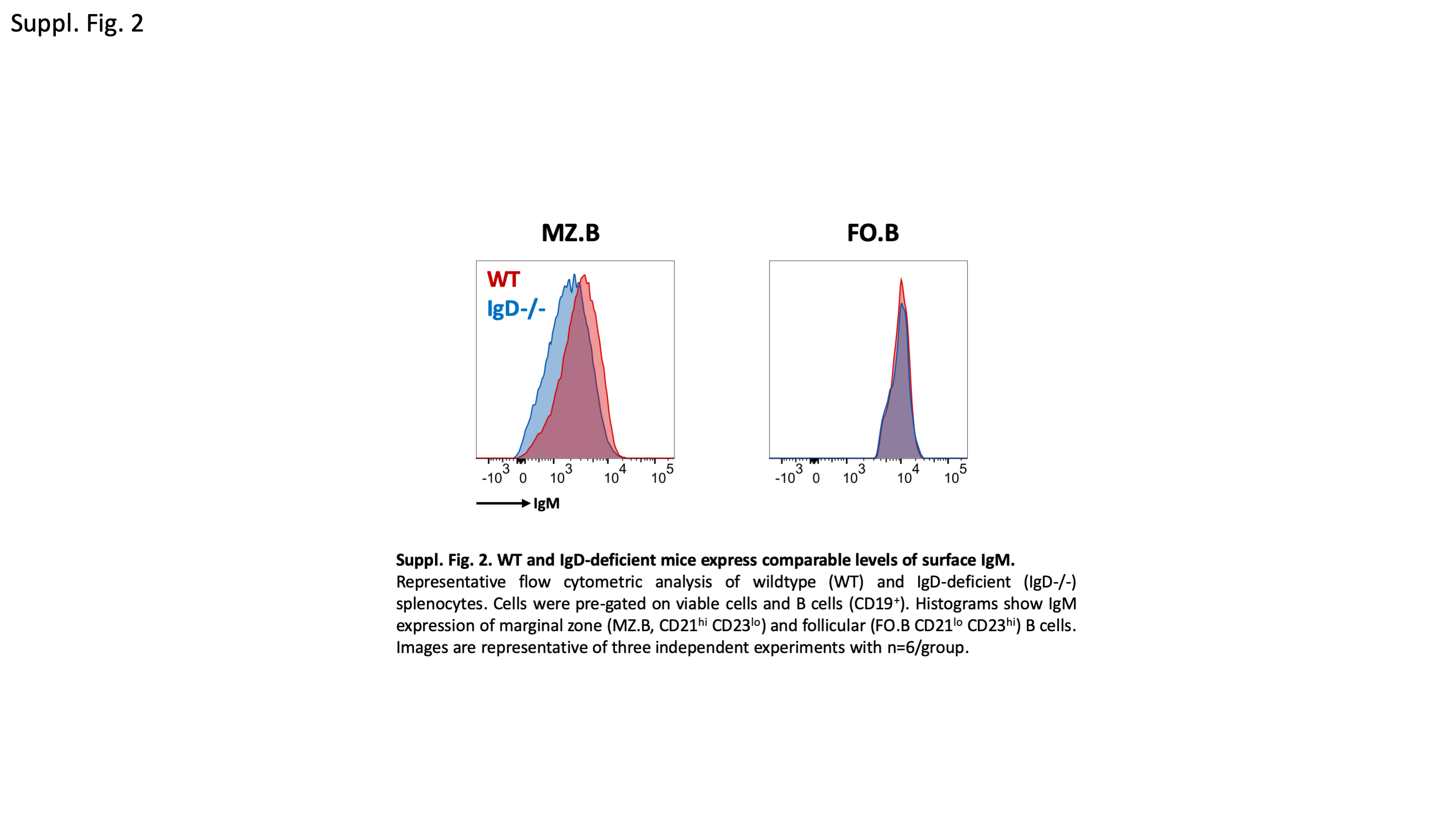

Supplement: Supplementary file 2 [file Image_2.tiff]

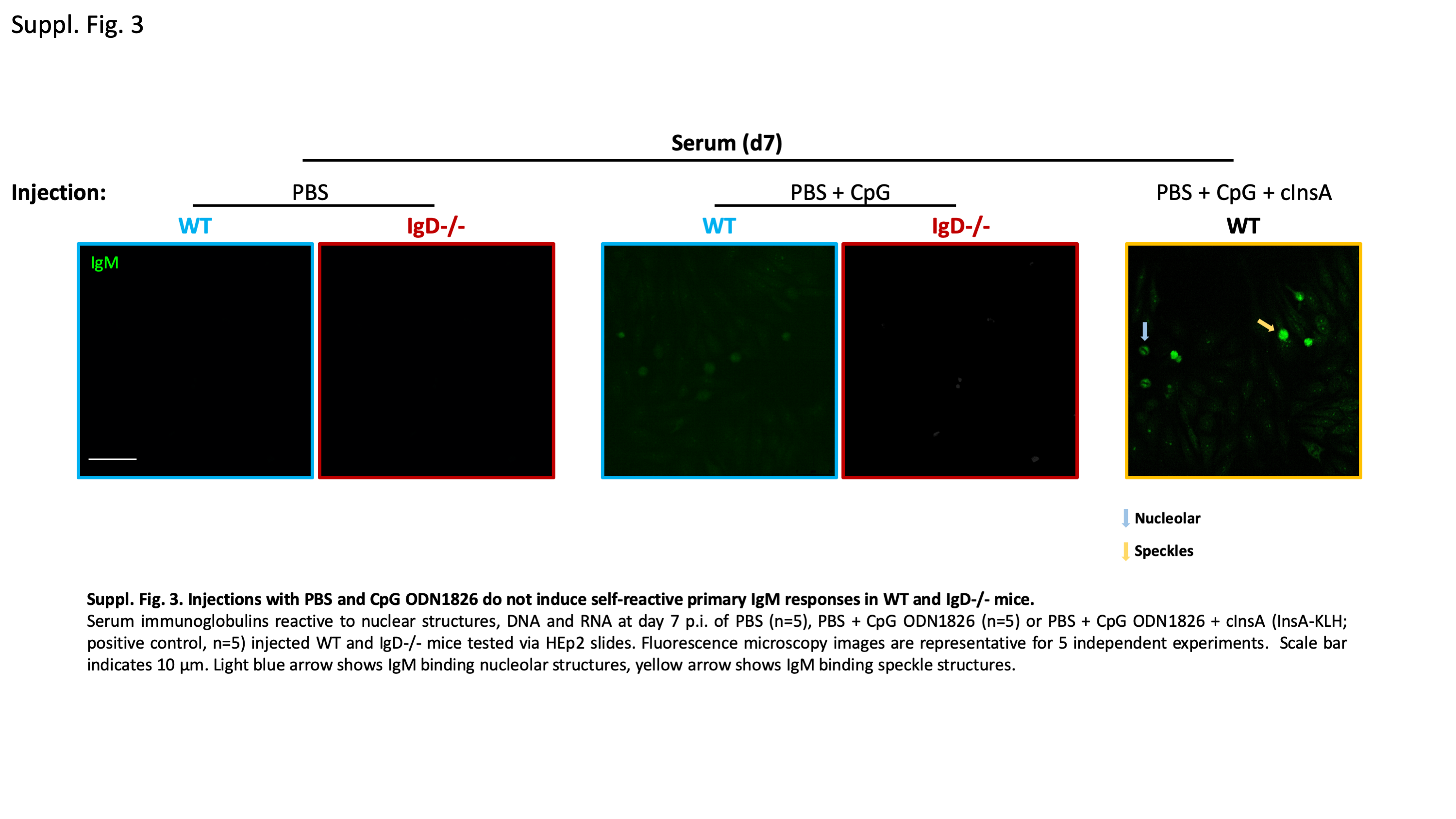

Supplement: Supplementary file 3 [file Image_3.tiff]

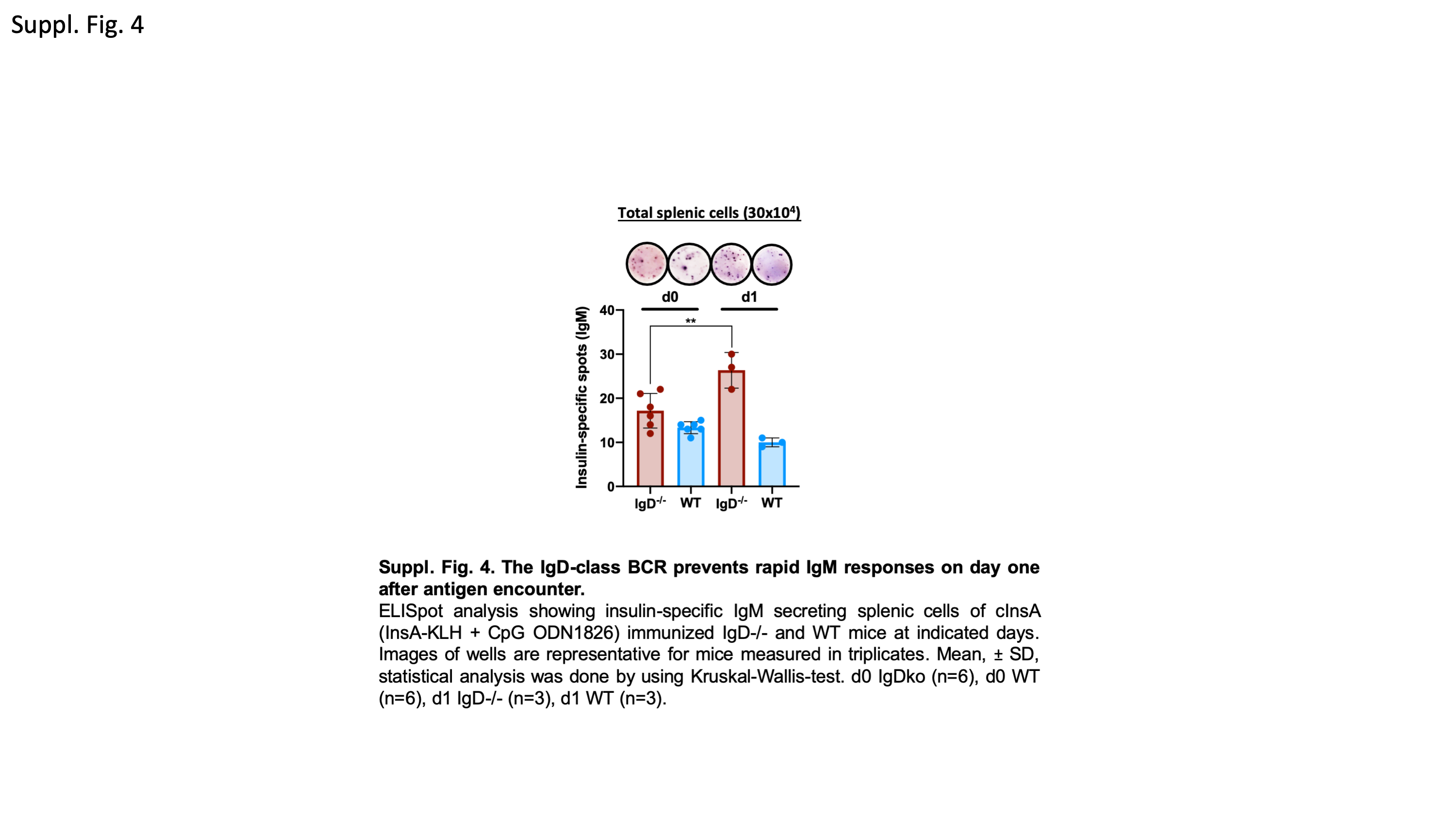

Supplement: Supplementary file 4 [file Image_4.tiff]

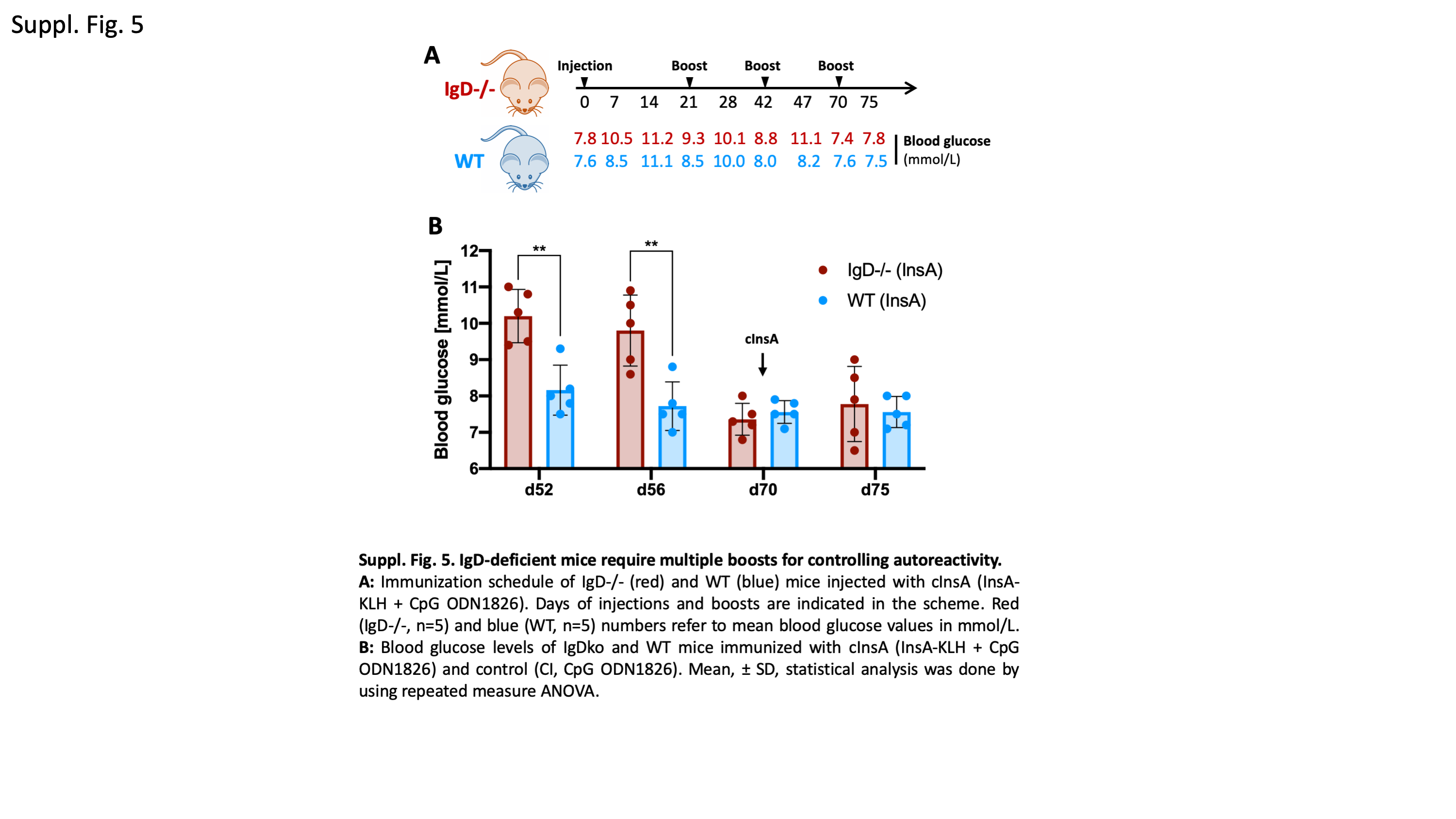

Supplement: Supplementary file 5 [file Image_5.tiff]

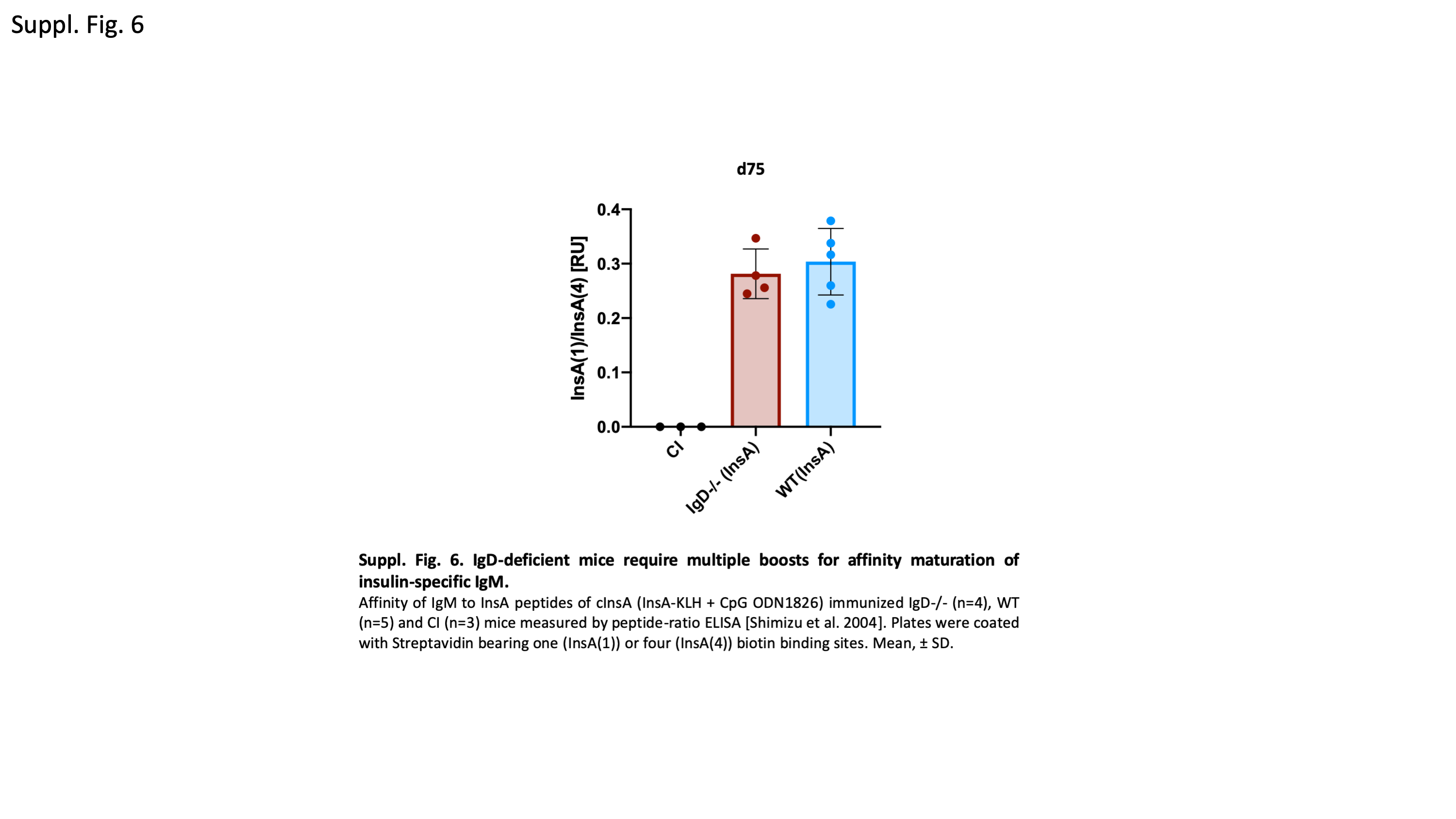

Supplement: Supplementary file 6 [file Image_6.tiff]

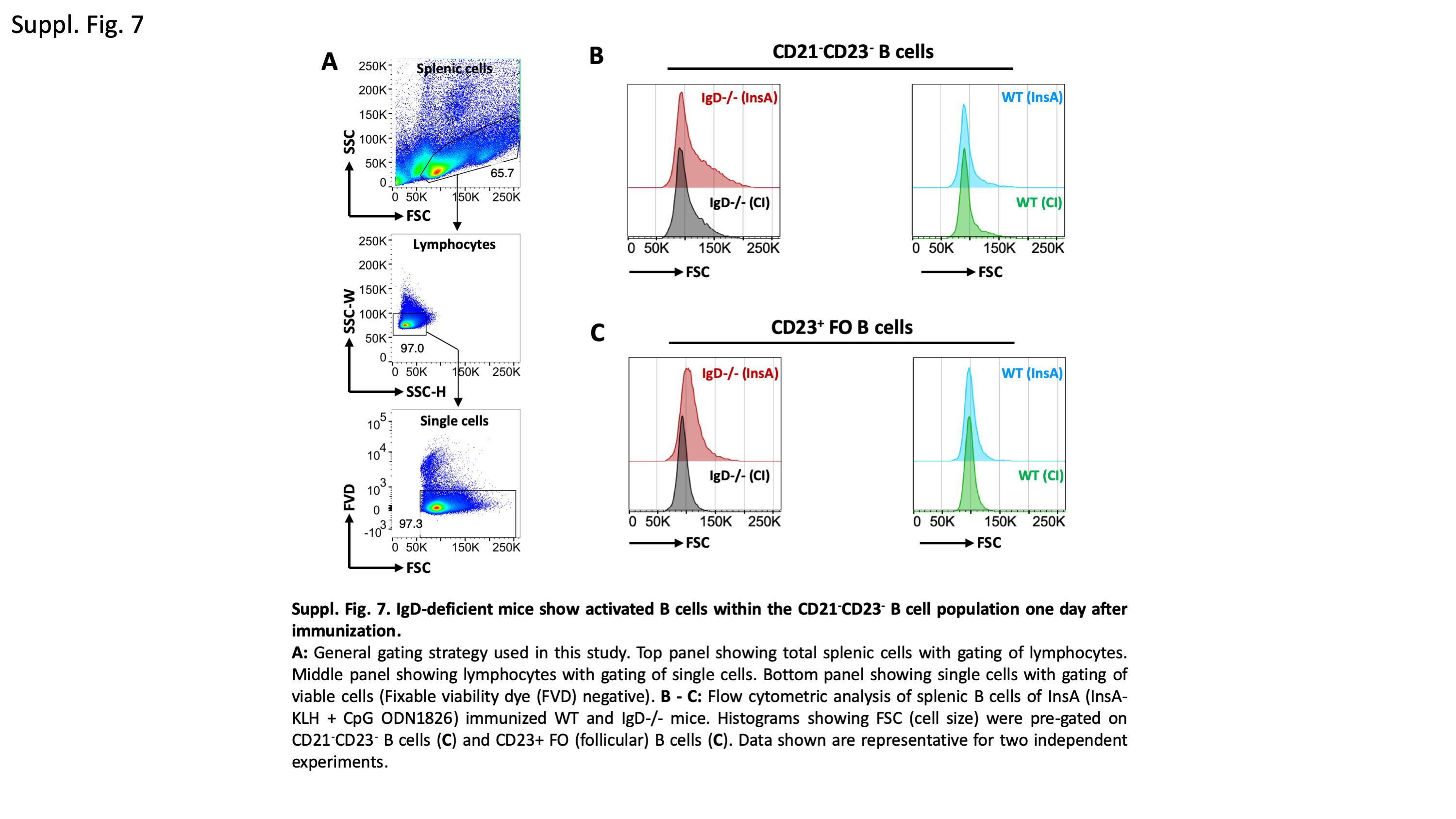

Supplement: Supplementary file 7 [file Image_7.tiff]

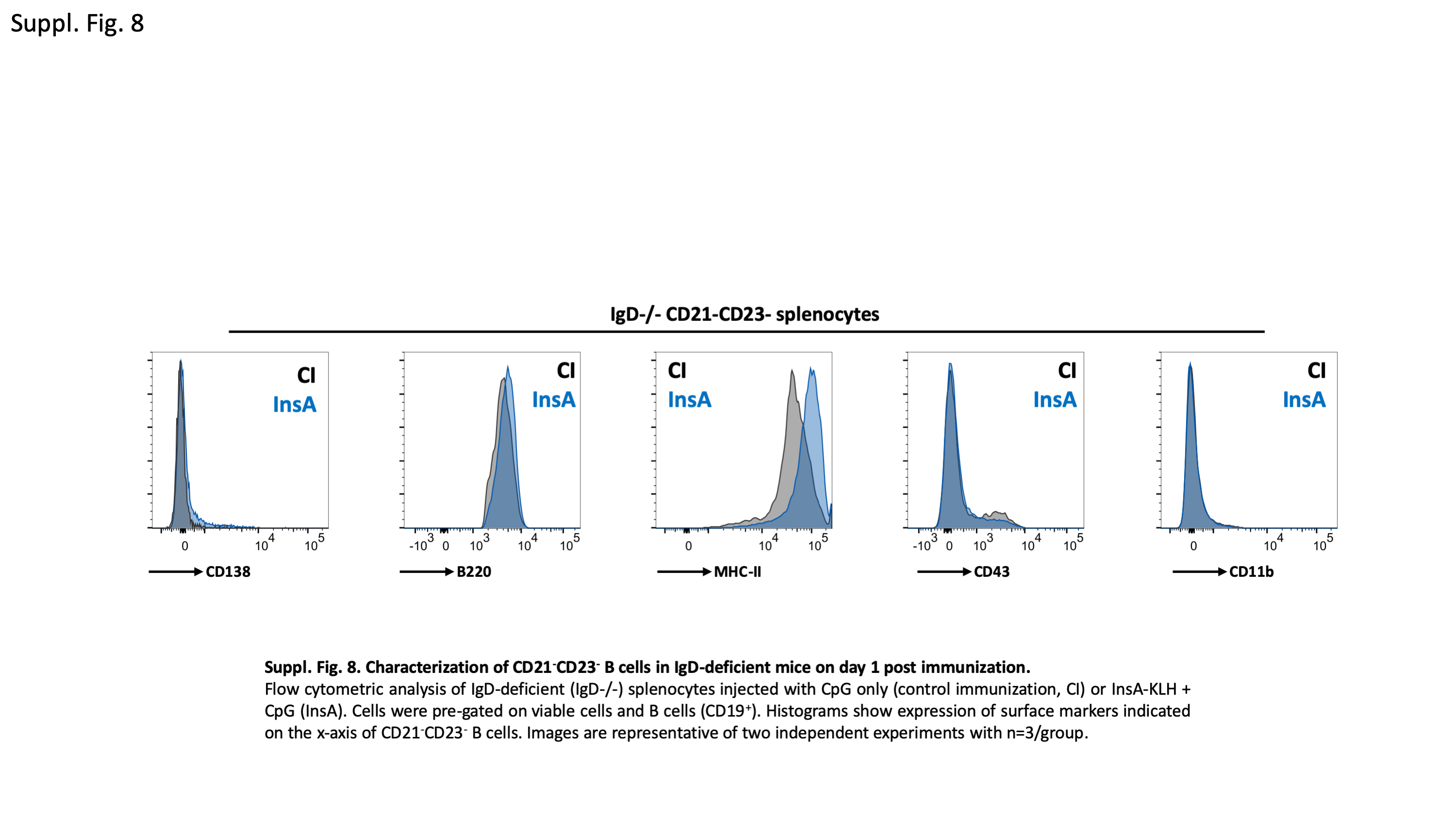

Supplement: Supplementary file 8 [file Image_8.tiff]

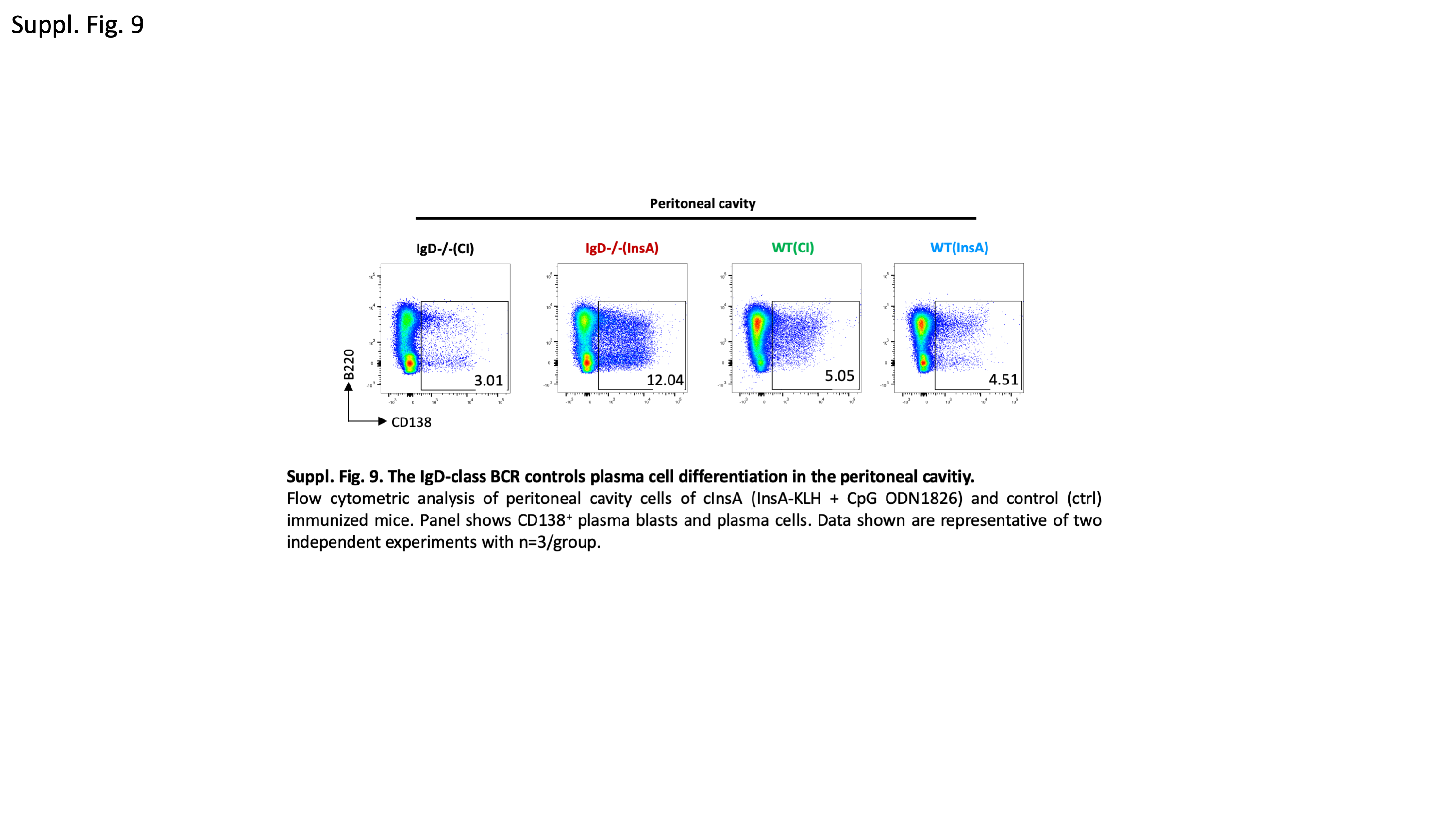

Supplement: Supplementary file 9 [file Image_9.tiff]
